# Supplementary material for: Administration of oral dosage forms of medicines to children in a resource limited setting
Source: PLoS One. 2022 Dec 22;17(12):e0276379. doi: 10.1371/journal.pone.0276379 (PMC9778530; doi:10.1371/journal.pone.0276379)
Supplement: S1 Table — (DOCX) [file pone.0276379.s001.docx]

Serial No

**Data collection sheet -Administration Indicators (Ward/OPD/Clinic)**

BHT number/ID No: DOB Age of the child: Weight of the child: Sex: M/F

Date and time of data collection: Setting

| **No** | **Drug which had a oral dosage form** | **Dose** | **Strength** | **Dosage form**  **Tablet/Capsule/Syrup/Suspension/**  **Drops** | **Swallowed whole**  **Yes/No** | **Manipulation**  **(Solid)** | | | | | | **Liquid measuring** | | | | **Water source** | | | **Repeat dose** | | | | | **Dose completion**  **(Yes/No)** | **Office use** |
| --- | --- | --- | --- | --- | --- | --- | --- | --- | --- | --- | --- | --- | --- | --- | --- | --- | --- | --- | --- | --- | --- | --- | --- | --- | --- |
|  |  |  |  |  |  | **Split** | **Crush** | **Open** | **Dissolved** | | | **Oral syringe** | **Others** | | | **Boiled cool** | **Tap** | **Others** | **Vomit** | **Spit**  **out** | **Mistakenly Measured** | **Others** | **Yes/No** |  |  |
|  |  |  |  |  |  |  |  |  | **Water** | **Milk** | **Others** |  | **Teaspoon** | **Measuring cup** | **Others** |  |  |  |  |  |  |  |  |  |  |
| 1 |  |  |  |  |  |  |  |  |  |  |  |  |  |  |  |  |  |  |  |  |  |  |  |  |  |
| 2 |  |  |  |  |  |  |  |  |  |  |  |  |  |  |  |  |  |  |  |  |  |  |  |  |  |
| 3 |  |  |  |  |  |  |  |  |  |  |  |  |  |  |  |  |  |  |  |  |  |  |  |  |  |
| 4 |  |  |  |  |  |  |  |  |  |  |  |  |  |  |  |  |  |  |  |  |  |  |  |  |  |
| 5 |  |  |  |  |  |  |  |  |  |  |  |  |  |  |  |  |  |  |  |  |  |  |  |  |  |
| 6 |  |  |  |  |  |  |  |  |  |  |  |  |  |  |  |  |  |  |  |  |  |  |  |  |  |

Collected
