# Supplementary material for: Administration of oral dosage forms of medicines to children in a resource limited setting
Source: PLoS One. 2022 Dec 22;17(12):e0276379. doi: 10.1371/journal.pone.0276379 (PMC9778530; doi:10.1371/journal.pone.0276379)
Supplement: Attachment — Submitted filename: Reponses to Reviewers comments .docx [file pone.0276379.s002.docx]

Dear Editor,

We would like to thank you and the reviewer for careful and thorough reading of this manuscript and for the comments and positive suggestions. Our response to each point raised are given below and the corrections are highlighted in the revised manuscript.

Thanking you.

Yours Sincerely,

Abarna Nadeshkumar

(Corresponding author)

**Response from authors to the comments**

1. **Reviewer’s Comment**: Thank you for allowing me to review this revised manuscript. While much improved there are some persistent areas that require attention and revision:
   1)Inclusion exclusion criteria (Line number 73 under methods)
   Inclusion criteria
   I. Prescriptions which had been prescribed with at least one oral dosage form of
   medicine within a month from the day of data collection to children under the age of 12
   years (dispensed may be a more definite word here vs prescribed?) Medication is the usual term vs medicine

**Author’s response:** Thank you for the comment. I have changed the word prescription into dispensing (Line number 74) and the word medicine in to medication (Entire manuscript)

2. **Reviewer’s Comment:** Parents/ guardians giving the data were the ones who gave medicines to the child at home (giving suggest change to providing)

**Author’s response:** Thank you for the comment. The wording has been changed to providing (Line number 76).

3. **Reviewer’s Comment**: Parents/ guardians should have the prescriptions with them at the time of data collection. (recommend change "should" to must to strengthen the inclusion criteria)

**Author’s response:** The wordings has been changed to must (Line number 78) .

4. **Reviewer’s Comment**: Exclusion criteria
   I. Prescriptions without oral dosage forms.
   II. Prescriptions issued. Older than one month (recommend change this sentence-doesn’t make sense in current form. Suggest something similar to "Prescriptions dispensed more than 30 days prior to data collection"

**Author’s response:** The recommended change has been done (Line number 82).

5. **Reviewer’s Comment:**  Please re-review the entire manuscript carefully for correct grammar and typos
   Again, suggest review by a native English speaker, perhaps not involved in the study to obtain a clear concise edited text
   There are persistent grammatical and a spelling error
   Please see lines 72, 194,200,206, 226,228 for revision

**Author’s response:** Entire manuscript was re reviewed for grammar and typos and the lines 74,200,205,211,233,235 were revised.

6. **Reviewer’s Comment**: With respect to regression analysis, this recommendation was rejected based on that this was not in the original study question, however it may strengthen the study. If you do not wish to run the regression to provide a robust analysis of this issue, then please discuss the need to explore factors predisposing to inappropriate administration of medications by parents to children in your discussion. Thank you.

**Author’s response:** Thank you for your comment. It has been discussed in the discussion (Line number 250).

7. **Reviewer’s Comment:**  Clearly state in the Discussion section exactly how information to parents/caregivers in a busy clinic with long wait times can be provided (printed information on posters, handouts, etc)

**Author’s response:** Thank you for your comment. It has been discussed in the discussion (Line number 237).

8. **Reviewer’s Comment** : Recommend stating clearly in a section on limitations that demographics, adherence and number of children in the household were not evaluated

**Author’s response:** Limitation has been clearly stated in the discussion now. (Line number 246).

9. **Reviewer’s Comment**: When writing about the incorrect use of medication dispensing devices and drugs of narrow therapeutic index, please specify the proportion of your study drugs where of this nature...for example in a regional referral center, children may receive anticoagulants, antiarryhmics, antiepileptics that require exact dosage administration

Finally, in the conclusion when stating issues, strongly recommend that some of these are stated...such as disease transmission, bioavailability, and over and under dosing. The current conclusion is rather weak.
Good luck in your final revisions!

**Author’s response:** Proportion of the study drug has been mentioned (Line numbers 145,155 .The conclusion has been rewritten. (Line number 252).
